# Saline irrigation versus gauze wiping and suction only for peritoneal decontamination during laparoscopic repair for perforated peptic ulcer disease

**DOI:** 10.1038/s41598-023-27471-0

**Published:** 2023-01-20

**Authors:** Lien-Cheng Tsao, Joseph Lin, Kuo-Hua Lin, Sze-Yuin Ng, Cheng-Yen Huang, Yu-Ju Hung, Szu-Chia Wu, Shih-Ling Gao, Shu-Fen Yu, Chi-Chien Lin, Wei-Jung Chang

**Affiliations:** 1 Department of General Surgery, Changhua Christian Hospital, Changhua, 500 Taiwan; 2 Institute of Biomedical Science, College of Life Sciences, National Chung-Hsing University, Taichung, 402 Taiwan; 3 Department of Animal Science and Biotechnology, Tunghai University, Taichung, 407 Taiwan; 4 Department of General Surgery, Yuanlin Christian Hospital, Yuanlin, 510 Taiwan; 5 Transplant Medicine and Surgery Research Center, Changhua Christian Hospital, Changhua, 500 Taiwan; 6 Department of Nursing, Changhua Christian Hospital, Changhua, 500 Taiwan; 7 Department of Medical Research, China Medical University Hospital, Taichung, 404 Taiwan; 8 Department of Pharmacology, College of Medicine, Kaohsiung Medical University, Kaohsiung, 807 Taiwan

**Keywords:** Gastrointestinal diseases, Medical research, Outcomes research

## Abstract

The aim of current single-center study was to compare the short-term outcome of suction and gauze wiping alone versus the irrigation and suction technique for peritoneal decontamination among patients who underwent laparoscopic repair of PPU. Using data from our institution’s prospectively maintained database, 105 patients who underwent laparoscopic repair were enrolled in this study. The participants were further divided into the group who received peritoneal irrigation (irrigation group, n = 67) and group who received gauze wiping and suction only (suction only group, n = 38). The irrigation group had a longer operative time (140 vs. 113 min, *p* = 0.0001), higher number of drainage tubes (38.8% vs. 0%, *p* < 0.0001) and a higher incidence of intra-abdominal abscess (10.4% vs. 0%, *p* = 0.0469) than the suction only group. Peritoneal irrigation may be associated with a prolonged operative time and a higher number of abdominal drains. Meanwhile, gauze wiping and suction may be sufficient for peritoneal decontamination during the laparoscopic repair of PPU as further infectious complications are not observed.

## Introduction

The incidence of perforated ulcer has decreased over recent years due to the identification of *Helicobacter pylori* and medical therapies being widely used thereafter. However, ulcer perforation continue to occur, with an incidence rate of up to 20%^1^. Surgical management is the definite treatment for patients with perforated peptic ulcers (PPU)^2^, and the laparoscopic approach for PPU repair is among the most common procedures performed by general surgeons^3,4^. Intra-abdominal abscess (IAA) is one of the frequently reported complications of laparoscopic repair for PPU, with an overall incidence rate of 4.4%^5^, which can be correlated with infectious abdominal contamination^6,7^. Peritoneal irrigation with saline solution, which is performed to dilute pollution, is mainly applied to decrease the incidence rate of postoperative IAA^8,9^. But there are no sufficient scientific data supporting the efficacy of this method^10^. Some studies have reported that peritoneal irrigation is associated with a higher risk of IAA after laparoscopic appendectomy^11,12^. Laparoscopic treatment of PPU has gained acceptance because it is correlated with a lower level of postoperative pain, incidence of wound-related complications, and shorter length of hospital stay^3,13^. However, due to the lack of consensus with minimal clinical evidence, the use of peritoneal lavage for decreasing the incidence of postoperative complications has been debated.

The aim of current single-center study was to compare the short-term outcome of suction and gauze wiping alone versus the irrigation and suction technique for peritoneal decontamination among patients who underwent laparoscopic repair of PPU. The research results shall improve surgical options with respect to risk and potential benefits in this setting.

## Methods

### Patients

In this retrospective study, we reviewed our institution prospectively maintained database and identified the adult patients with a clinical diagnosis of PPU who underwent emergent surgery between January 2013 and July 2021. The preoperative diagnosis of PPU was based on typical images showing the presence of pneumoperitoneum, intraperitoneal fluid, and discontinuity of the gastrointestinal wall. Patients with severely unstable hemodynamic condition, previous history of laparotomy surgery, malignant ulcer, concomitant ulcer bleeding, and coagulopathy were not eligible for laparoscopic surgery and were, thus, excluded from the analysis. Patients who underwent laparoscopic repair were included. A complete chart review was conducted to determine whether the patient had irrigation performed intraoperatively which consisted of normal saline solution and suctioned out subsequently. Patients who did not have irrigation had visible purulent or peritoneal exudate suctioned out followed by gauze wiping. Then, the participants were divided into the group who received intraoperative peritoneal irrigation and suction (irrigation group) and group who received intraoperative gauze wiping and suction without irrigation (suction only group) for decontamination. In both groups, the pre- and postoperative managements were similar. Preoperatively, all patients were treated with nasogastric tube decompression, intravenous fluid resuscitation, and intravenous broad-spectrum empiric antibiotics according to the Surviving Sepsis Campaign^14^. Postoperatively, they continually received management with intravenous PPIs, pain medications, and chest physiotherapy. If the condition is uneventful, the patients were started on water and oral diet on postoperative day 1, with gradual transition to oral pain medications and antibiotics until discharge. During the clinical follow-up, upper gastrointestinal endoscopy was performed 1 month after discharge. Patients continually received oral PPIs with additional triple therapy for *Helicobacter pylori* eradication if the *Campylobacter*-like organism test or the stool antigen test had positive results. The primary outcome of interest was postoperative complication including IAA, wound infection, post-repair leakage, and postoperative pneumonia. IAA was defined as a complicated intra-abdominal fluid collection in the setting of fever ≥ 38 °C, abdominal pain or an elevation of inflammatory marker levels. Leakage was defined as evident gastroenteric or feeding content in the drainage bulb. The secondary outcomes were operative time, volume of blood loss, ICU admission rate, number of drainage tubes, and length of hospital stay. This study was approved by the institutional review board of Chanhua Christian Hosptial (CCH IRB No: 201021). Written informed consent was waived by the ethics board due to the retrospective nature of this study. This study was also registered to ClinicalTrials.gov (NCT05147870).

### Surgical intervention

**Figure 1:**
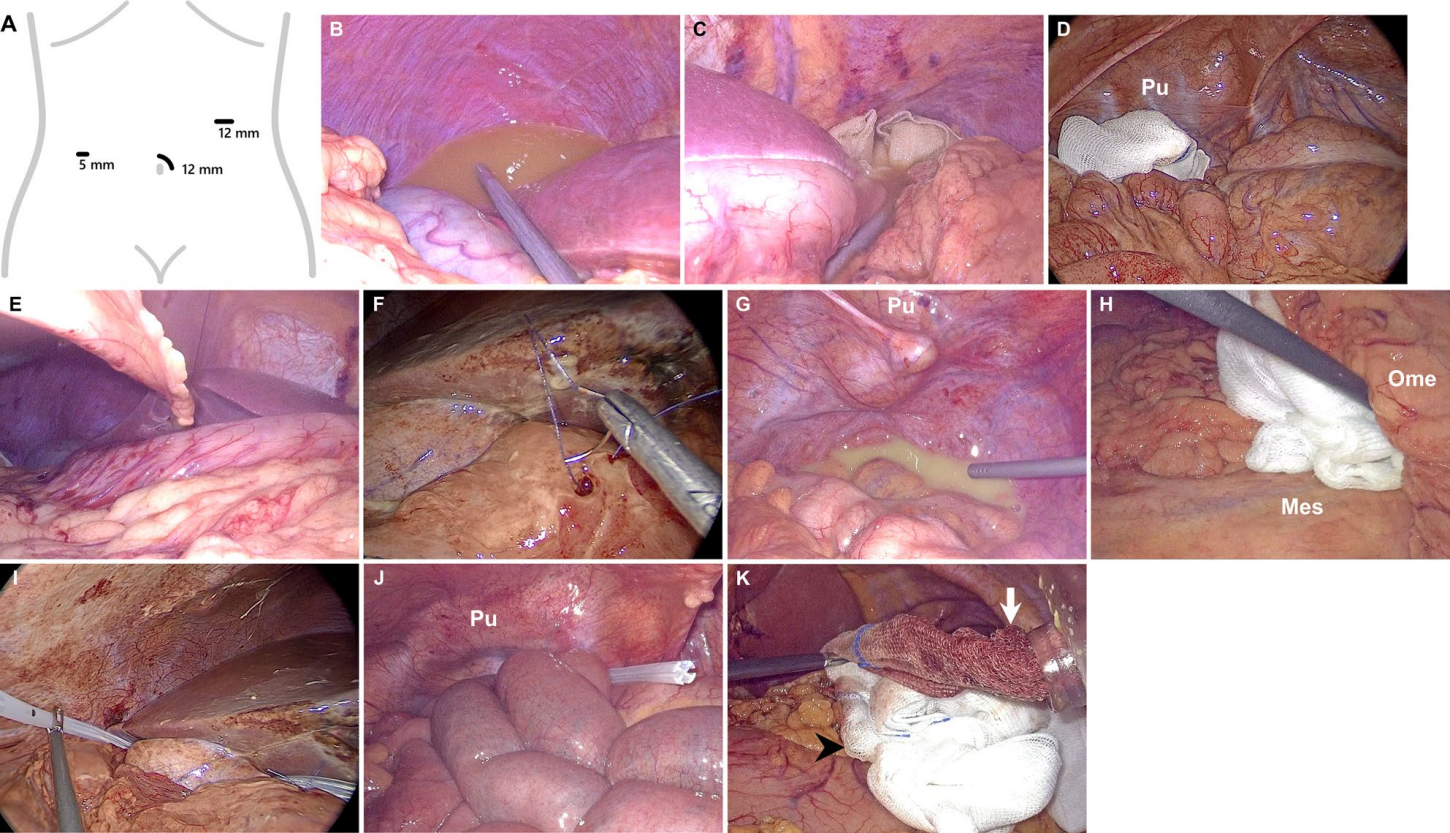
Schematic drawing and intraoperative images during laparoscopic repair of PPU. (**A**) Schematic drawing showing the establishment of ports. The three-port method was used, with a periumbilical camera port (12 mm), one working port (12 mm) in the left midclavicular line, and the third port (5 mm) in the right abdominal region. After the initial exploration of the peritoneal cavity, purulent collections over (**B**) the Morrison’s pouch and (**C**) the left subphrenic area were evacuated with a laparoscopic suction instrument. (**D**) Purulent fluid at the Cul-de-sac was evacuated, and a surgical gauze was then placed for further fluid absorption before the patients were placed into the reverse Trendelenburg position. (**E**) The transabdominal suture traction of the round ligament with a 2-O straight PROLENE was applied to elevate the liver to improve the exposure of the pyloroduodenal region. (**F**) A two-layer, simple interrupted suture with 3-O Vicryl plus was used to repair the perforated site. (**G**) One surgical gauze was placed in the splenophrenic space and the Morison’s pouch to soak up any remaining purulent fluid. Next, the patient was turned into the Trendelenburg position to assess the lower abdominal cavity. (**H**) Any interloop adhesions were cautiously divided, and the gauze wiping maneuver was used to soak up the residual peritoneal fluid. All bowel loops were investigated to the root of the mesentery. Drainage tubes were placed at the dependent area of the abdomen cavity such as the Morrison pouch (**I**) and the left subphrenic area or Cul-de-sac (**J**). (**K**) Before pulling out any dirty and soaked gauze (white arrow), we siphoned off as much excess fluid as possible using the suction device. Then, a clean gauze (black arrowhead) was carpeted underneath the trocar to take up surplus fluid as the moist gauze passed and squeezed through the laparoscopic trocar. *Ome* omentum, *Mes* mesentery, *Pu* pubic bone.

We performed simple closure routinely, which was an effective, safe, and time-conserving procedure applied in previous studies^4,15^. Patients were placed in the supine position with arms tucked to the sides. A nasogastric tube and Foley’s urinary catheter were used to decompress the stomach and bladder, respectively. All patients received antibiotic treatments upon anesthesia induction or admission in the emergency department where the diagnosis was made. Three-port laparoscopic repair was performed under general anesthesia. In general, a periumbilical incision was made. Then, a 12-mm camera port was established using the Hasson technique. A 30° laparoscope was then introduced. The 12-mm left upper quadrant working trocar was then placed in the left midclavicular line to facilitate the insertion of sutures and surgical gauzes. The third 5-mm right abdominal working port was placed slightly cranial to the camera port (Fig. 1A). After the initial exploration of the peritoneal cavity, purulent collections were evacuated with a laparoscopic suction instrument (Fig. 1B,C). Surgical gauze was then positioned at the pouch of Douglas before the patients were placed into the reverse Trendelenburg position (Fig. 1D). The transabdominal suture traction of the round ligament with a 2-O straight PROLENE was applied to elevate the liver (Fig. 1E). This could facilitate a better exposure of the pyloroduodenal region where the perforation could be meticulously searched and identified. Both the Morison’s pouch and the splenophrenic space were inspected, and peritoneal fluids were further evacuated.

After the perforation site was identified, the perforation size was assessed with reference to the size of the jaw of the laparoscopic grasp. Simple interrupted suturing with 3-*O* Vicryl plus was conducted to repair the perforated site (Fig. 1F). Patients in the irrigation group received peritoneal lavage according to the surgeon’s discretion based on surgical findings and preference. In general, the operating table was tilted appropriately to facilitate exposure, and warm saline was used for irrigation from quadrant to quadrant to obtain adequate lavage.

In the suction only group, surgical gauze was placed each in the splenophrenic space and the Morison’s pouch to soak up any remaining purulent fluid. Next, the patient was turned into the Trendelenburg position to evaluate the lower abdominal cavity (Fig. 1G). Any interloop adhesions were cautiously divided. The gauze wiping maneuver was used to soak up the residual peritoneal fluid, and all bowel loops up to the root of the mesentery were investigated (Fig. 1H). In both groups, the soaked gauze was then pulled out via the 12-mm port, and each gauze soaked 10 mL of ascitic fluid. All patients received drainage tubes placed at the dependent parts of the abdominal cavity such as the Morrison pouch, left subphrenic area, and pouch of Douglas (Fig. 1I,J). The number and location of drainage tubes however were dependent on the contaminated severity of the abdominal cavity and the decision of the individual surgeon. Additionally, before pulling out any dirty and soaked gauze (white arrow), we siphoned off as much excess fluid as possible using the suction device. Then, a clean gauze (black arrowhead) was carpeted underneath the trocar to take up surplus fluid as the moist gauze passed and squeezed through the laparoscopic trocar (Fig. 1K). We inserted a new gauze through the trocar to wipe off any remaining fluid inside the trocar valves.

### Statistical analysis

The Mann–Whitney U test was used to compare clinical scale and ordinal variables, and they were expressed as median and interquartile range (IQR). Nominal variables were compared using the chi-square test or the Fisher’s exact test. Statistical analyses were performed using MedCalc for Windows (version 20; MedCalc; Ostend, Belgium). A *p* value of < 0.05 was considered statistically significant.

## Results

From October 2012 to July 2021, 702 patients presented to the emergency room, and they were clinically diagnosed with PPU and underwent surgery in our hospital. In total, 597 patients had surgery using the open approach. Among them, 21 underwent laparoscopic surgery initially. However, the procedure was then intraoperatively converted to laparotomy because the perforation site could not be repaired or was challenging to identify or approach. The remaining 105 patients who underwent laparoscopic repair were enrolled in this study. The participants were further divided into two groups: that is, 67 patients in the irrigation group and 38 in the suction only group. The clinical profile of the patient cohort is shown in Table 1. The mean age was 59.4 years, and there was a male predominance (70.5%). The Boey score was used to assess postoperative risks. In total, 48 (45.7%) patients had a score of 0; 45 (42.9%), 1; and 12 (11.4%), 2. The Mannheim peritonitis index was employed to assess the severity of peritonitis, and the population under study was categorized into mild (score: < 21), moderate (score: 21–29), and severe (score: > 29)^16,17^. Altogether, 23 (21.9%), 64 (61%), and 18 (17.1%) patients were classified into the mild, moderate, and severe groups, respectively. A total of 23 (21.9%) and 82 (78.1%) patietns were classified as the American Society of Anesthesiology (ASA) score of 1–2 and 3–4 respectively. Overall, no significant differences were found between the two groups in terms of age (*p* = 0.0641), sex distribution (*p* = 0.1538), preoperative pulse rate (*p* = 0.8025), body temperature (*p* = 0.8465), systolic blood pressure (*p* = 0.3732), white blood cell count (*p* = 0.4412), Boey score (*p* = 0.2162), Mannheim peritonitis index (*p* = 0.6496), and ASA classification (*p* = 0.6859). Only one patient in the irrigation group received additional feeding jejunostomy. During the postoperative follow-up, 30 (33.0%) patients underwent testing for *Helicobacter pylori* infection. Among them, four tested positive and 26 negative.

**Table 1:** Patient characteristics at diagnosis of perforated peptic ulcer.

	Irrigation (n = 67)	Suction only (n = 38)	*p* value
Age, years	58.0 (44.5–69.0)	64.5 (50.0–74.0)	0.0641
Male gender, n (%)	44 (65.7)	30 (78.9)	0.1538
**Vital signs on arrival**			
Temperature, °C	36.7 (36.1–37.0)	36.7 (36.2–37.1)	0.8465
Pulse, beats/min	88.0 (75.0–100.0)	90.0 (73.0–101.0)	0.8025
SBP, mmHg	129.0 (118.0–150.0)	135.0 (118.0–154.0)	0.3732
WBC, × 1000/mm^3^	12.3 (8.6–17.2)	11.5 (7.4–15.6)	0.4412
**ASA score, n (%)**			0.6859
1, 2	16 (23.9)	7 (18.4)	
3, 4	51 (76.1)	31 (81.6)	
**Boey score, n (%)**			0.2162
0	33 (49.3)	15 (39.5)	
1	29 (43.3)	16 (42.1)	
2	5 (7.5)	7 (18.4)	
**Mannheim peritonitis index, n (%)**			0.6496
Mild (< 21)	16 (23.9)	7 (18.4)	
Moderate (21–29)	41 (61.2)	23 (60.5)	
Severe (> 29)	10 (14.9)	8 (21.1)	

*ASA* The American Society of Anesthesiology.

*p < 0.05.

Perioperative data was showed in Table 2. The irrigation group had a longer operative time than the suction only group (140 vs. 113 min, *p* = 0.0001). All patients had abdominal drainages. In total, 28 (26.7%) patients had one drain; 51 (48.6%), two; and 26 (24.8%), three. The irrigation group had a higher number of patients with three drainage tubes than the suction only group (38.8% vs. 0%, *p* = 0.0001). The volume of intraoperative blood loss in both groups was low, and there was no statistically significant difference between the two groups (15.7 vs. 8.6 mL, *p* = 0.098). Further, the perforation size (9.7 vs. 9.6 mm, *p* = 0.956) and ascites volume (200.0 vs. 300 mL, *p* = 0.2911) did not differ significantly between the two groups. The irrigation group had a significantly higher volume of saline solution, ranging from 7000 to 10,000 mL, than the suction only group.

**Table 2:** Perioperative outcomes.

	Irrigation (n = 67)	Suction only (n = 38)	*p* value
Operating time, min	140 (125–175)	113 (95–140)	0.0001*
**Perforation site**			0.9903
Stomach, n (%)	23 (34.3)	13 (34.2)	
Duodenum, n (%)	44 (65.7)	25 (65.8)	
Perforation size^a^, mm	9.7 ± 5.5	9.6 ± 8.4	0.956
Ascites volume, ml	200 (100–500)	300 (100–550)	0.2911
Blood loss^a^, ml	15.7 ± 25.0	8.6 ± 9.67	0.098
Irrigation volume, ml	9500 (7000–10,000)	0 (0–0)	< 0.0001*
**Drainage number**			< 0.0001*
1, n (%)	22 (32.8)	6 (15.8)	
2, n (%)	19 (28.4)	32 (84.2)	
3, n (%)	26 (38.8)	0 (0)	

*p < 0.05.

^a^Represented as mean value ± SD.

The postoperative outcomes and surgical complications were evaluated using the Clavien–Dindo classification^18^ (Table 3). The incidence rate of IAA was 6.7% (n = 7). One patient with IAA required radiological intervention, and the remaining patients received pharmacological treatments. The repair site leakage rate was 3.8% (n = 4). Among patients who had site leakage, three underwent duodenorrhaphy with reinforcement of the previous suture site using laparotomy methods. Moreover, they received intensive care unit management, and their recovery was uneventful. The fourth case was a 61-year-old patient (Boey score 1) who had diabetes and chronic kidney disease stage IV and who was recently diagnosed with brain tumor died from septic shock and multiple organ dysfunction secondary to repair site leakage. The family members of this patient refused further salvage intervention.

**Table 3:** Postoperative complications.

	Irrigation (n = 67)	Suction only (n = 38)	*p* value
Hospital mortality, n (%)	1 (1.5)	2 (5.3)	0.2963
**Wound infection, n (%)**	0 (0)	1 (2.6)	0.3619
Grade I	–	1	
**Pneumonia, n (%)**	6 (9.0)	8 (21.1)	0.1325
Grade II	2	5	
Grade IV	3	2	
Grade V	1	1	
**Leakage, n (%)**	2 (3.0)	2 (5.3)	0.6192
Grade III	–	1	
Grade IV	2	–	
Grade V	–	1	
**IAA, n (%)**	7 (10.4)	0 (0)	0.0469*
Grade II	6	–	
Grade III	1	–	
ICU admission, n (%)	13 (19.4)	13 (34.2)	0.0927
Hospital stay, days	8 (7.0–10.8)	9 (7.0–17.0)	0.0454*

Complication grade according to Clavien–Dindo Classification.

*IAA* Intraabdominal abscess.

*p < 0.05.

There were no significant differences in the rate of wound infection (0% vs. 2.6%, *p* = 0.3619), leakage rate (3.0% vs. 5.3%, *p* = 0.6192), and postoperative pneumonia (9.0% vs. 21.1%, *p* = 0.1460). However, the incidence rate of IAA was significantly lower in the suction only group than in the irrigation group (0% vs. 10.4%, *p* = 0.0469). Although the length of hospital stay significantly differed between the two groups (8 vs. 9 days, *p* = 0.0454), it has minimal clinical impact. Three patients died in the postoperative period (irrigation group: n = 1, suction only group: n = 2). All patients presented with ASA III disease. Besides the above mentioned 61-year-old male died from septic shock secondary to repair site leakage, the other two were aged over 75 years. The patients were as follows: First, an 82-year-old man (Boey score 1) with a history of hypertension, chronic obstructive pulmonary disease, and chronic kidney disease stage III in the irrigation group died of multiorgan failure secondary to sepsis. Second, a 75-year-old female patient (Boey score 2) with diabetes, nephropathy, and hypertension underwent PPU surgery while on chemotherapy for metastatic breast cancer. She then refused further organ supportive therapy and died after 8 days due to sepsis and multiorgan failure.

## Discussion

This is the first study to assess the influence of peritoneal irrigation on surgical outcomes after laparoscopic repair of PPU. The current study demonstrated that the suction-only group was associated with a shorter operative time (113 vs. 140 min, *p* = 0.0001), a lower incidence rate of IAA (0% vs. 10.4%, *p* = 0.0469), and a lesser number of drainage lines (*p* < 0.0001). The annual incidence of PPU, ranged from 0.004 to 0.014%, decreased during the 1990s and early 2000s^19–22^, which resulted from the treatment of *Helicobacter pylori* with the combination of antibiotics and a proton pump inhibitor (PPIs)^23^. However, it still requires emergency surgery^24^. Previous reports revealed that laparoscopic repair is safe and reliable for treating PPU^3,25,26^. Compared to open repair, laparoscopic method involved a shorter length of hospital stay, less postoperative pain and reduced overall morbidity^3,13,27,28^. Laparoscopic PPU management can provide a clear and magnified surgical view of the peritoneal cavity compared with the traditional subxiphoid-umbilicus incision, but whether the laparoscopic method can aspirate all the fluid collected between the interloop spaces and bilateral subphrenic areas remains unknown. A recent meta-analysis revealed that the IAA rates were similar between the open and laparoscopic PPU repair methods, which may imply a comparable clearance of purulent ascites along with the irrigated solution at the end of surgery regardless of the method used (open or minimally invasive)^28^.

In our institution, we performed simple closure routinely, which was an effective, safe, and time-conserving procedure applied in previous studies^4,15^. The literature revealed an overall leakage rate after laparoscopic repair of 6.3%^29^. In our series, four patients experienced leakage (two in the irrigation group and two in the suction only group), and the incidence rate was 3.8%, which was lower than that of previous reports. However, the low repair site leak rate does not reflect the simplicity of surgery itself nor the superiority of the repair method. Rather, it might be attributed to the fact that the procedures of patients with a large perforation and/or technical difficulties were converted to the open method after the initial laparoscopic exploration.

The post-repair PPU surgical mortality rate ranges from 1.3 to 20% regardless of the method used (open vs. laparoscopic)^5^. Three (2.86%) patients in our study died, which falls within the lower end of the previous reports. This is likely attributed, in part, to the fact that only 11 (10.5%) patients with ASA IV risk were included in this study. Previous studies showed that the laparoscopic repair of PPU may be associated with more favorable outcomes in terms of postoperative pain, length of hospital stay, and recovery to performing daily activities^3,13,27,28^. However, these results might be biased due to the selection of younger patients, lower ASA scores, and earlier access to the emergency department after the onset of symptoms^5^. Based on the current study, 93 (88.6%) patients had a low Boey score (0 or 1).

Peritoneal irrigation has been considered a reasonable treatment option for septic abdominal diseases for many years^8,9^. This concept was raised from the notion that “dilution is the solution to pollution.” However, peritoneal lavage with fluid accumulation might pose a potential risk of subsequent infection, particularly in a contaminated abdomen, and we assumed that the postoperative IAA rate can reflect the residual fluid collection. Data about its efficacy in reducing postoperative morbidity and mortality rates in this context are extremely limited. A randomized and prospective trial assessed the clinical value of peritoneal lavage with saline or antibiotic solution and compared it with the suction-only method, which did not show a lower incidence of infectious complications after surgical treatment for abdominal sepsis^30^. A previous experimental animal study revealed a high volume of retained intraperitoneal irrigation solution and a poor mortality rate^31^. This was associated with the dilution of opsonins secondary to the retained irrigation solution, which attenuated the leukocyte phagocytosis mechanism^32^. Therefore, suctioning of all irrigated solutions is more effective than the historical-based lavage itself.

To date, no study has assessed the knowledge and efficacy of peritoneal irrigation against postoperative infectious complications in cases of PPU during laparoscopic repair. However, a few studies have reported that peritoneal irrigation is associated with a high risk of IAA after laparoscopic appendectomy^12,33^. A meta-analysis conducted by Gammeri et al. demonstrated that there is no evidence of benefit of lavage over suction in the prevention of IAA after appendectomy^34^. Irrigation can possibly be harmful due to the following reasons: First, it may cause diffuse inoculation and spread contamination particularly into the dependent areas (such as the pelvis and subphrenic space) where retrieval can be challenging using a suction probe. Second, it does not decrease the microorganism load in the peritoneum. Third, it may further dilute the mediators of phagocytosis that fight against infections^32^. By contrast, Sun et al. performed a prospective randomized trial. Results showed that copious irrigation of the peritoneal cavity could be an effective method for lowering the incidence of postoperative IAA in laparoscopic appendectomy for complicated appendicitis compared with suction alone (3.1% vs. 9.2%, *p* = 0.039)^35^. The use of surgical gauze was applied to facilitate dissection, organ retraction and clearing blood and intra-abdominal fluids. Moreover, the use of it also avoids inadvertent aspiration of the omentum or intestinal wall into the suction device and reduces the loss of pneumoperitoneum. In this study, we also determined the added value of gauze wiping to soak up the remaining purulent fluid and remove the fibrin film coating on the peritoneal cavity. The non-use of irrigation was not associated with a higher rate of IAA in our patients. Therefore, the removal of purulent effluent, rather than dilution and irrigation, is important. Furthermore, there is no consensus regarding the adequate volume of fluid that should be used for irrigation. Bertleff et al. performed a review of 29 studies that used warm saline with a volume ranging from 2 to 6 L for lavage. However, some studies have reported irrigation with up to 10 L^29^.

In the current study, we compared morbidity and mortality rates to assess two different techniques. Among these parameters, IAA could be an indicator of residual fluid collection after PPU surgery. With respect to the safety of laparoscopic repair for PPU, the overall IAA rate is 4.4%^5^. Based on our series, seven patients presented with IAA, and the incidence rate was 6.7%. The incidence rate of IAA was significantly lower in the suction only group than in the irrigation group (0% vs. 10.4%, *p* = 0.0469). In the suction only group, the use of surgical gauze in the peritoneal cavity facilitated aspiration as it prevented inadvertent aspiration of the omentum or intestinal wall into the suction device, which may result in bleeding or serosal tear. Moreover, a gauze can soak up the remaining purulent effluent and is effective in removing the fibrin film coated on the peritoneal cavity, bowel, and visceral surfaces^36^. However, the non-use of irrigation was not associated with a higher rate of IAA in our patients. Hence, the removal of purulent effluent, rather than dilution and irrigation, is important.A previous systemic review compared the wound infection rate associated with laparoscopic repair versus open repair for perforated peptic ulcer, and it was significantly lower in laparoscope group with a reported rate of 2.2%^28^. From our study, the overall wound infection rate was 0.95%, which was lower than that of previously studied. This might be explained due to the retrospective nature of this study in which the presence of wound infection was based on medical record documentation and medication prescription. Only one case was recorded with umbilical wound seroma and none of these patients needed surgical intervention for wound. However, we could not neglect the fact that the trocar might be protective of the incisional wound during the surgery from the contaminated environment and dirty gauzes.

The current study showed that, in contrast to the suction with irrigation, suction only is associated with a shorter operative time (140 vs. 113 min, *p* = 0.0001), and a fewer number of drains were inserted (*p* < 0.0001). The longer operative time in the irrigation group could be attributed to peritoneal irrigation. That is, an earlier report showed that peritoneal lavage was correlated with a longer operative time than perforated ulcer suturing^37^. The drain placement was designed to prevent fluid collection and subsequent infection, and the number of drainage placements depended upon the surgeons’ individual preference and specific clinical judgment. We used gauze to wipe out the contaminated content and postoperative drains were placed to monitor the postoperative condition in the suction-only group. Mostly, we needed one or two drains in the Morison’s pouch (also besides the perforation site) and the pouch of Douglas. The surgeons might be worried about the residual fluid after irrigation in the group; therefore, we inserted more drains. The drains were placed in the Morison’s pouch, pouch of Douglas, and left subphrenic areas to avoid fluid collection. A study showed significantly greater pain scores (measured by visual analog scale) in the drain group after laparoscopic cholecystectomy compared to the non-drain group, though pain and life quality of reduced drains was not mentioned^38^. Our study revealed less number of drains after the gauze wiping and suction method with similar outcomes. We could not assess the pain score and quality of life regarding the number of drains due to its retrospective nature. However, these two factors will be assessed in the prospective cohort study that we are currently conducting, and their association necessitates a further report.

The current study had a few limitations. First, this was a retrospective, non-randomized, single-institution study with a relatively small sample size. We selected the subgroup suitable for laparoscopic surgery after excluding the patients with a severely unstable hemodynamic condition, previous history of laparotomy surgery, malignant ulcer, concomitant ulcer bleeding, and coagulopathy. Although 702 patients were initially diagnosed with PPU, selection bias attributed to this study could have existed. The lower rate of laparoscopic surgery in the current study may be explained by that our hospital is the only tertiary referring hospital draining four governorates, therefore a good portion of those 579 patients were referred and admitted with Boey score 2 or 3. Hence, the entire population has been preselected in a way that a higher proportion of PPU patients with severe clinical conditions were presented to our hospital. Furthermore, we could not assess any significant predictors of IAA after laparoscopic repair, which might be attributed to the sample size with an insufficient power. However, the low incidence of IAA indicates that even though a statistically significant difference can be achieved by increasing the sample size, this difference may not be translated into a clinically meaningful consequence in real-world settings. Moreover, there were no significant differences between the two groups in terms of preoperative patient characteristics (Table 1). Similarly, the operative time was influenced by several factors such as skill of different surgeons, methods of wound closure, and impact of the experience of the first assistant surgeon, which was challenging to quantify in this study. This study benefits from its real-world clinical data and a relatively standardized strategy for surgical approach. Currently, we are conducting a prospective cohort study with a larger sample size to further evaluate the impact of the use of irrigation for peritoneal decontamination after laparoscopic repair of PPU, and we hope to share our results soon.

The importance of irrigation during laparoscopic repair for PPU remains unknown. To the best of our knowledge, this was the first study that compared the postoperative outcomes of two different cleansing methods for the abdominal cavity. Results showed that peritoneal irrigation might be associated with a higher incidence of postoperative IAA and number of abdominal drains and prolonged operative time. Meanwhile, the gauze wiping and suction only method maybe adequate for peritoneal decontamination during laparoscopic repair for PPU.

## Data Availability

The data that support the findings of this study are available from the institutional review board of Changhua Christian Hospital but restrictions apply to the availability of these data, which were used under license for the current study, and so are not publicly available. Data are however available from the authors upon reasonable request and with permission of the institutional review board of Changhua Christian Hospital.
